# Supplementary material for: A Global Screen for Assembly State Changes of the Mitotic Proteome by SEC-SWATH-MS
Source: Cell Syst. 2020 Feb 26;10(2):133–155.e6. doi: 10.1016/j.cels.2020.01.001 (PMC7042714; doi:10.1016/j.cels.2020.01.001)

Q5H9R7 | PP6R3_HUMAN | PPP6R3 C11orf23 KIAA1558 PP6R3 SAPL SAPS3
Monomer MW [kDa]: 97.669 Monomer expected elution fraction: 40

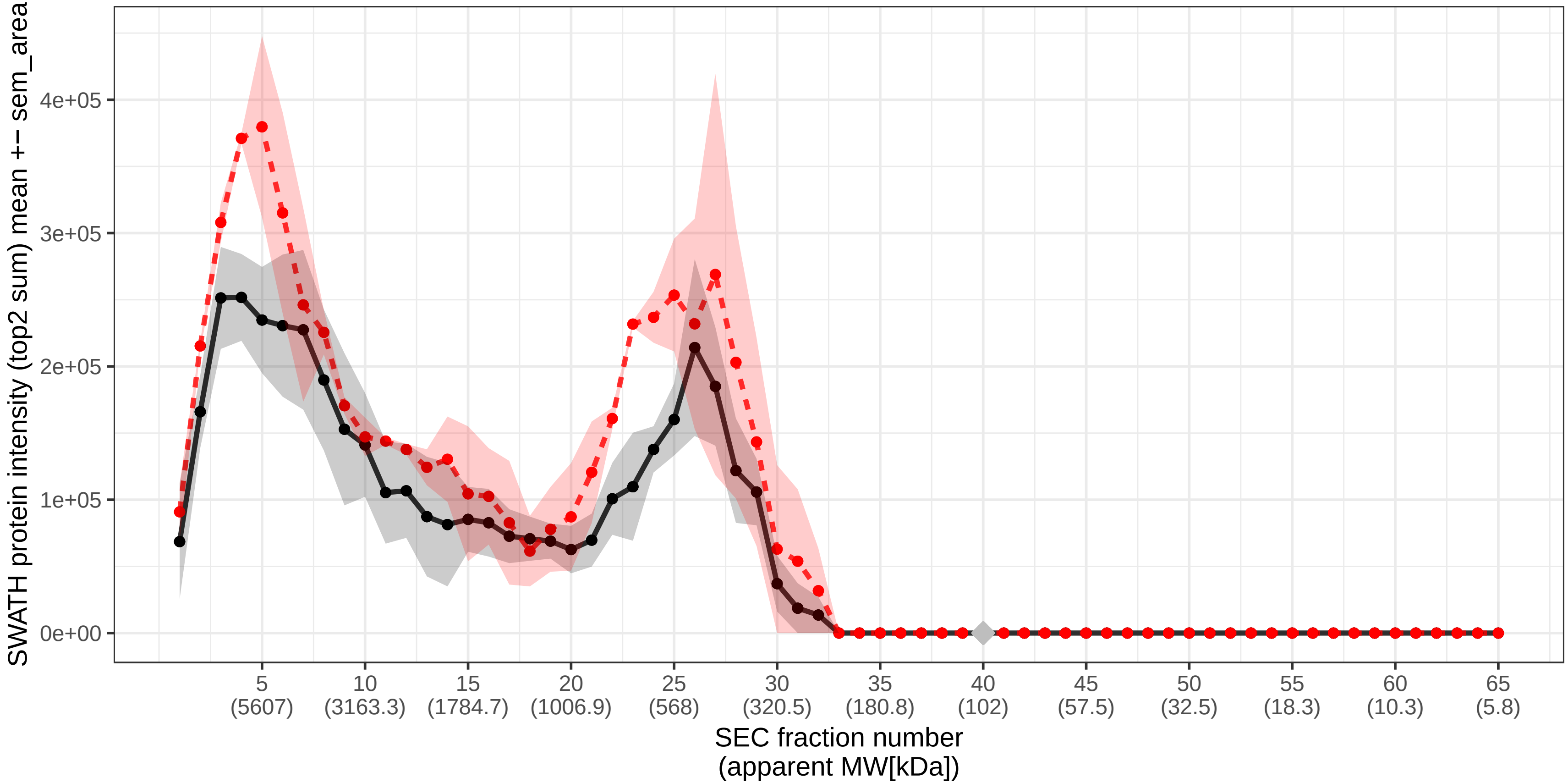

Supplement: Data S1. SEC-SWATH-MS Protein Chromatograms, Related to Figure 1 [file mmc6.zip › SECchrom_Q5H9R7_PP6R3_HUMAN_PPP6R3_C11orf23_KIAA1558_PP6R3_.pdf]
